# Datasets for the effects of RUNX2 silencing on transcriptomic and metabolomic profiles in SJSA-1 osteosarcoma cells

**DOI:** 10.1016/j.dib.2023.109500

**Published:** 2023-08-18

**Authors:** Mai Nhu Uyen Le, Ruiqi Chen, Liang-e Xia, Jianlin Zhou, Yichong Ning

**Affiliations:** a State Key Laboratory of Developmental Biology of Freshwater Fish & Key Laboratory of Protein Chemistry and Developmental Biology of the Ministry of Education, College of Life Science, Hunan Normal University, Changsha, Hunan 410081, China; b Hunan Key Laboratory of Tumor Models and Individualized Medicine, The Second Xiangya Hospital, Central South University, Changsha, Hunan, China; c Chongzuo Key Laboratory of Biomedical Clinical Transformation, The People's Hospital of Chongzuo, Youjiang Medical University for Nationalities, Chongzuo, Guangxi, China

**Keywords:** Bone tumor, RNA interference, RNA-seq, Metabolic reprogramming, Runt-related transcription factor

## Abstract

Osteosarcoma is the most common primary malignant bone tumor with a high risk of metastasis and recurrence. Metabolic reprogramming is a hallmark of osteosarcoma and other cancers and is associated with genetic and epigenetic alterations. RUNX2 is an important transcription factor for osteoblastic differentiation, and aberrant expression of the gene contributes to the development and progression of osteosarcoma. To identify the effects of RUNX2 silencing on transcriptomic and metabolomic profiles in osteosarcomas, we generated SJSA-1 osteosarcoma cells stably expressing RUNX2 shRNA and SJSA-1 cells stably expressing scramble shRNA and analyzed transcriptome and metabolome profiles in the two cell types using Illumina NovaSeq 6000 and ultrahigh-performance liquid chromatography coupled with time-of-flight mass spectrometry, respectively. The datasets can be used by researchers to identify novel targets of RUNX2 and elucidate the role and underlying mechanism of RUNX2 in osteosarcoma pathogenesis and metabolic reprogramming.

Specifications Table

**Table:** 

Subject	Cancer research
Specific subject area	Transcriptomics and metabolomics
Type of data	Tables, MS files
How the data were acquired	RNA-seq data were acquired by Illumina NovaSeq 6000. Untargeted metabolomic data were acquired by the ultrahigh-performance liquid chromatography coupled with time-of-flight mass spectrometry.
Data format	Raw and processed data
Description of data collection	The SJSA-1 human osteosarcoma cells stably expressing RUNX2 shRNA or scramble shRNA were constructed. Total RNA was extracted using TRIzol reagent. Paired-end RNA libraries were prepared and sequenced using standard Illumina protocols. Illumina Casava1.7 software was used for base calling. Sequenced reads were trimmed for adaptor sequence, and masked for low-complexity or low-quality sequence, then mapped to the Homo sapiens GRCh38.87 whole genome. Gene expression was calculated as fragments per kilobase of transcript per million mapped reads (FPKM) using featureCounts. Metabolites were extracted with a methanol-acetonitrile solution under sonication twice for 30 min. Chemicals were separated using Agilent 1290 Infinity LC ultrahigh-performance liquid chromatography (UHPLC) system with HILIC chromatography column, followed by positive and negative electrospray ionization (ESI) analyzes (Agilent 6550). Metabolites were identified using AB Triple TOF 6600 mass spectrometer and the first-order QC samples were collected. Raw data were converted to mzXML format using ProteoWizard and then aligned using the XCMS program. Peak area between correction and extraction. Metabolite structures were identified by searching the metabolite database using precision mass-number matching (<25 ppm) and second-order map.
Data source location	Hunan Normal University, 36 Lushan Road, Changsha, China
Data accessibility	The raw and processed RNA-seq data were deposited at the Gene Expression Omnibus (GEO) database with the identifier GSE158976 (https://www.ncbi.nlm.nih.gov/geo/query/acc.cgi?&acc=GSE158976). The metabolome data were deposited at the China National GeneBank Database (CNGBdb) with the identifier CNP0004506 (https://db.cngb.org/search/project/CNP0004506).

## 1 Value of the Data

• The datasets present the effects of RUNX2 silencing on the gene expression pattern and metabolite profile in SJSA-1 osteosarcoma cells.
• The transcriptome dataset can be used by researchers to identify novel targets of RUNX2 and elucidate the molecular mechanism of RUNX2 in osteosarcoma tumorigenesis.
• The metabolome dataset can be used by researchers to elucidate the role of RUNX2 in metabolic reprogramming in osteosarcoma.
• The transcriptome and metabolome may be useful for integrated analysis to reveal the link between gene expression alteration and metabolic reprogramming.

## 2 Objective

Runt-related transcription factor 2 (RUNX2) is considered a crucial transcriptional regulator of skeletal development [1]. There is increasing evidence that abnormal expression of RUNX2 is a driving factor in osteosarcoma oncogenesis [2,3]. However, the molecular mechanism of RUNX2 remains unclear. The objective for the generation of these RNA-Seq and metabolome datasets was to identify the effects of RUNX2 silencing on the gene expression pattern and metabolite profile in osteosarcoma cells and provide raw data. The datasets will be useful for researchers to uncover the role and underlying mechanism of RUNX2 in osteosarcoma.

## 3 Data Description

SJSA-1 cells stably expressing scramble shRNA (shNC) and SJSA-1 cells stably expressing RUNX2 shRNA-1 (sh1) were generated, and six samples each were subjected to transcriptome and metabolome analysis (Table 1).

**Table 1:** General overview of samples described in this work.

Short name	Sample type
shNC-1	SJSA-1 cells overexpressing scramble shRNA, replicate 1
shNC-2	SJSA-1 cells overexpressing scramble shRNA, replicate 2
shNC-3	SJSA-1 cells overexpressing scramble shRNA, replicate 3
shNC-4	SJSA-1 cells overexpressing scramble shRNA, replicate 4
shNC-5	SJSA-1 cells overexpressing scramble shRNA, replicate 5
shNC-6	SJSA-1 cells overexpressing scramble shRNA, replicate 6
sh1-1	SJSA-1 cells overexpressing RUNX2 shRNA, replicate 1
sh1-2	SJSA-1 cells overexpressing RUNX2 shRNA, replicate 2
sh1-3	SJSA-1 cells overexpressing RUNX2 shRNA, replicate 3
sh1-4	SJSA-1 cells overexpressing RUNX2 shRNA, replicate 4
sh1-5	SJSA-1 cells overexpressing RUNX2 shRNA, replicate 5
sh1-6	SJSA-1 cells overexpressing RUNX2 shRNA, replicate 6

**Fig. 1:**
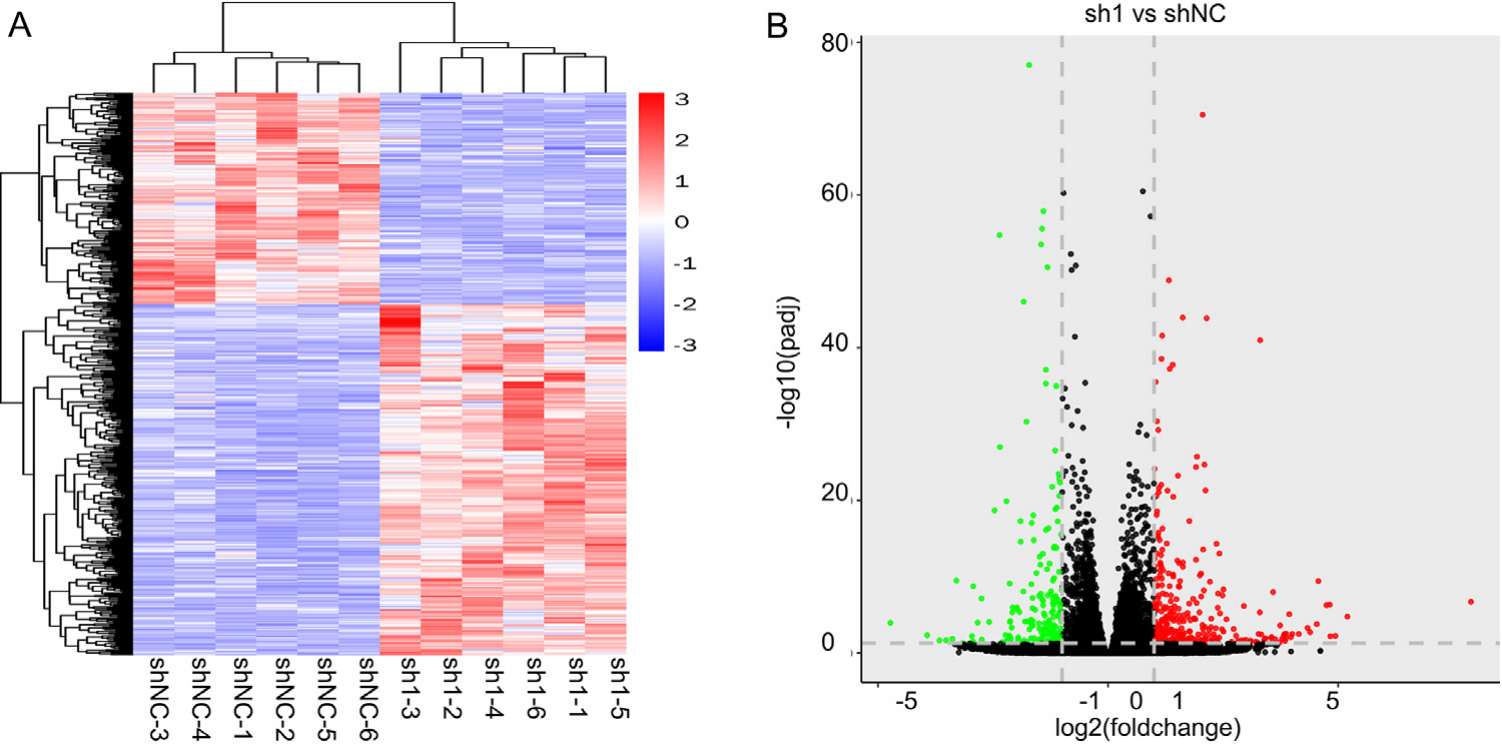
Heatmap (A) and volcano (B) plots show the differentially expressed genes between SJSA-1 cells stably expressing scramble shRNA-1 (shNC) and SJSA-1 cells stably expressing RUNX2 shRNA-1 (sh1-1).

Raw and processed RNA-seq data were deposited in the GEO database [4] under the identifier GSE158976. Table 2 shows the raw reads, clean reads and Phred quality scores of the RNA-seq data for all samples. Gene expression was calculated as FPKM using featureCounts [5]. Heatmap and volcano plots show the differentially expressed genes between SJSA-1 cells stably expressing scramble shRNA and SJSA-1 cells stably expressing RUNX2 shRNA-1 (Fig. 1), with 186 downregulated genes (log2FoldChange≤-1 and padj < 0.05) and 309 upregulated genes (log2FoldChange≥1 and padj < 0.05).

**Table 2:** Raw reads, clean reads and Phred quality scores of RNA-seq for all samples.

Sample	Raw_reads	Clean_reads	Error (%)	Q20(%)	Q30(%)
sh1-1	41186722	40698170	0.03	97.64	93.32
sh1-2	43809628	43314208	0.03	97.67	93.34
sh1-3	42117630	41602470	0.03	97.46	92.88
sh1-4	44173058	43788922	0.03	97.96	94.07
sh1-5	41931460	41546134	0.03	97.86	93.78
sh1-6	40066386	39708828	0.03	97.94	93.97
shNC-1	48791574	48369334	0.02	98.1	94.38
shNC-2	51092496	50572212	0.03	97.85	93.81
shNC-3	53527414	53017078	0.03	97.94	93.96
shNC-4	40671694	40296244	0.03	97.84	93.71
shNC-5	45849132	45412636	0.03	97.92	93.96
shNC-6	42514114	42063100	0.03	97.92	93.93

Note: Q20 (%): the percentage of bases in the reads with a phred quality ≥ 20; Q30 (%): the percentage of bases in the reads with a phred quality ≥ 30.

**Fig. 2:**
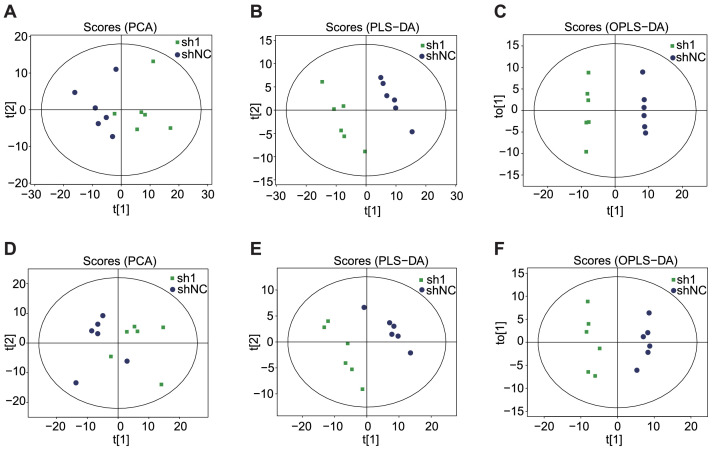
PCA, PLS-DA and OPLS-DA score plots in different groups under positive-ion (A, B, C) and negative-ion (D, E, F) modes.

**Fig. 3:**
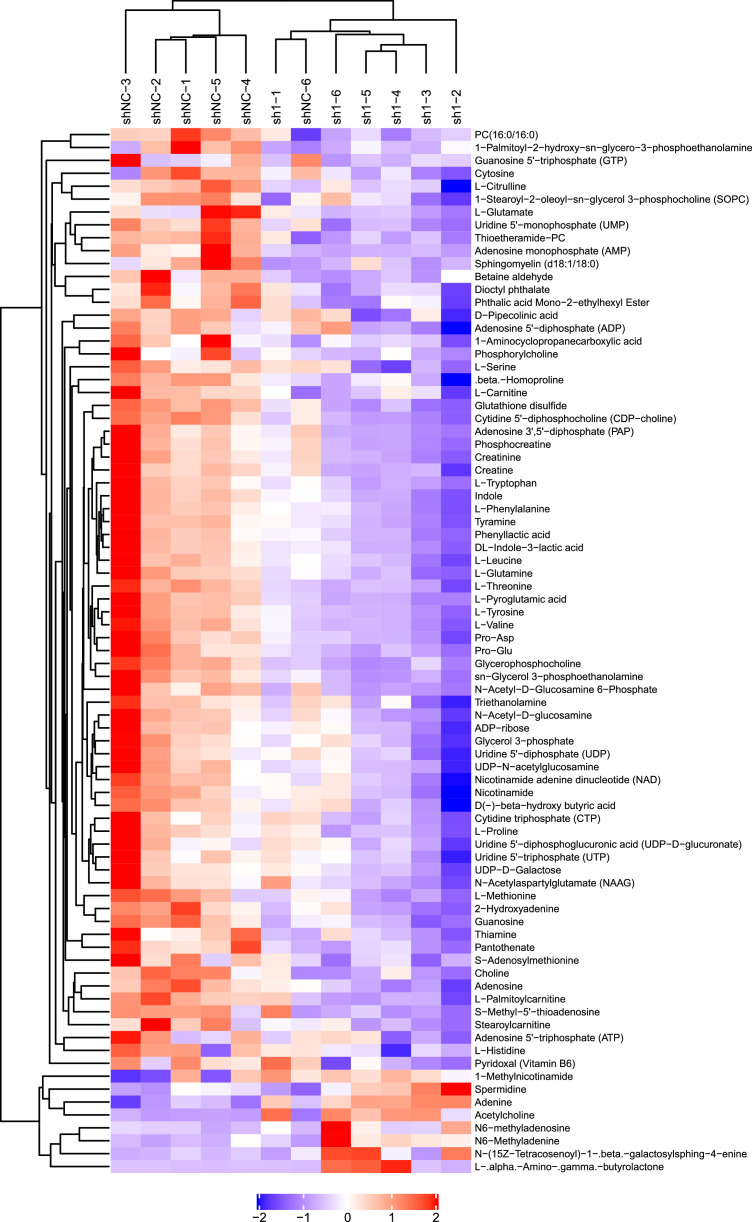
Heatmaps show the hierarchical clustering results of metabolites with significant differences between the sh1-1 and shNC groups under positive-ion mode.

**Fig. 4:**
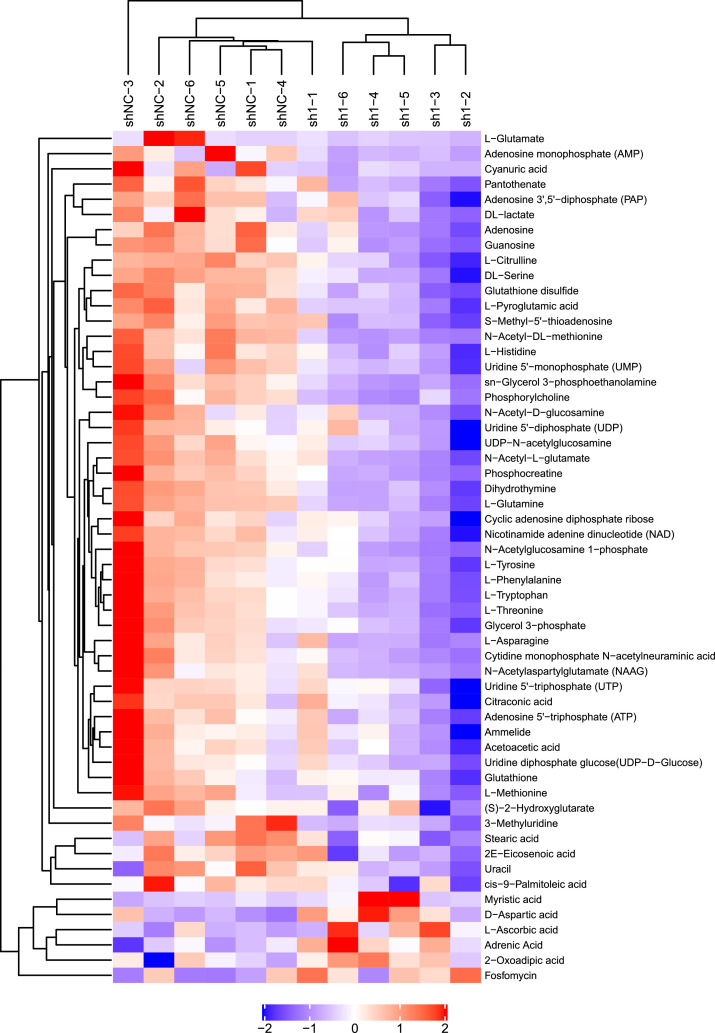
Heatmaps show the hierarchical clustering results of metabolites with significant differences between the sh1-1 and shNC groups under negative-ion mode.

Metabolome data were deposited in the CNGBdb database [6] with the identifier CNP0004506. Multidimensional statistical analysis, including principal component analysis (PCA), partial least squares discrimination analysis (PLS-DA) and orthogonal PLS-DA (OPLS-DA), showed that the overall metabolites displayed significant differences between the two groups (Fig. 2). Permutation tests showed that the PLS-DA and OPLS-DA models had good reliability and predictability (Table 3). The variable importance for projection (VIP) value of the OPLS-DA model and p value were used to screen differentially expressed metabolites (VIP value > 1 and *p* value < 0.05). Heatmaps show the results of hierarchical clustering of metabolites with significant differences between the two groups (Figs. 3 and 4).

**Table 3:** Permutation test parameters for PLS-DA and OPLS-DA models.

	Positive-ion mode			Negative-ion mode		
Model	R^2^X(cum)	R^2^Y(cum)	Q^2^(cum)	R^2^X(cum)	R^2^Y(cum)	Q^2^(cum)
PLS-DA	0.500	0.999	0.886	0.354	0.970	0.510
OPLS-DA	0.500	0.999	0.779	0.354	0.970	0.553

## 4 Experimental Design, Materials and Methods

### 4.1 Cell Culture, Lentiviral Transduction and Stable Cell Selection

The SJSA-1 osteosarcoma cell line was obtained from ATCC (Manassas, VA, USA) and cultured in RPMI-1640 medium supplemented with 10% newborn calf serum, penicillin (100 U/ml) and streptomycin (100 lg/ml) at 37°C in a 5% CO_2_ incubator according to ATCC's instructions. Lentiviruses expressing RUNX2 shRNA or scramble shRNA (shNC) were purchased from GeneChem (Shanghai, China). Three shRNAs targeting RUNX2 (sh1, sh2 and sh3) and nontargeting shNC were designed. Their target sequences are as follows: sh1: 5’-CAGCACTCCATATCTCTAC-3’, sh2: 5’-GTGGTCCTATGACCAGTCT-3’, sh3: 5’-TGCACTATCCAGCCACCTT-3’, and shNC: 5’-TTCTCCGAACGTGTCACGT-3’. Double-stranded DNA containing target and antisense sequences linked by a short spacer was cloned and inserted into the lentivirus vector GV248 (hU6-MCS-ubiquitin-EGFP-IRES-puromycin) to generate lentiviral particles. It was validated that sh-1 was most effective in suppressing RUNX2 expression and was used for this study. Cells were infected with lentiviral particles at a multiplicity of infection (MOI) of 8 in the presence of polybrene. At 72 h postinfection, infected cells were cultured in fresh medium with 2.5 µg/mL puromycin (Selleck Chemicals LLC, Houston, TX, USA) until the percentage of EGFP-positive cells reached 100%.

### 4.2 RNA Isolation, Library Construction, Sequencing, and Data Analysis

Total RNA was extracted using TRIzol reagent (Invitrogen, Carlsbad, CA, USA). The sequencing library was prepared using the NEBNext® Ultra™ RNA Library Prep Kit for Illumina (NEB, Ipswich, MA, USA) according to the manufacturer's instructions. Briefly, the mRNA was enriched with oligo(dT) beads. Fragmentation buffer was then added to randomly break the mRNA. The first strand of cDNA was synthesized with random hexamer primers, and then the second strand was synthesized with buffer, dNTPs, and DNA polymerase I. The purified double-stranded cDNA was then end-fixed, A-tailed and linked to the sequencing adapter, and the fragment size was selected using AMPure XP beads (Beckman Coulter, Brea, CA, USA), followed by PCR enrichment to obtain the final cDNA library. The library was subjected to 2 × 150 bp paired-end sequencing on the Illumina NovaSeq 6000 (San Diego, CA, USA). Raw image data were converted to raw sequence reads (sequenced reads) by Illumina's CASAVA software (version 1.8), which we refer to as raw data, and stored as FASTQ (Fq) files. Clean data were obtained by trimming off adaptor sequences and removing low-quality reads. The clean reads were mapped to the Homo sapiens GRCh38.87 whole genome using HISAT2 software [7]. Gene expression was calculated as FPKM using featureCounts [5].

### 4.3 Metabolite Detection and Analysis

Cells were cultured in 10-cm plates, washed with ice-cold PBS and ice-cold saline (0.9% NaCl solution), and then mixed with a methanol/acetonitrile/water mixture (2:2:1, v/v). Cells were collected in a 1.5 ml centrifuge tube, vortexed for 60 s, sonicated at -20°C for 2 × 30 min, and centrifuged at -4°C for 20 min. The supernatant was frozen and dried. Metabolites were separated using the Agilent 1290 UHPLC system (Santa Clara, CA, USA), followed by positive and negative ESI analyses (Agilent 6550) and time of flight (TOF) mass spectrometry (AB Sciex Triple TOF 6600, Framingham, MA, USA). Raw data were converted to mzXML format using the ProteoWizard tool [8] and processed for peak alignment, retention time correction and peak area using the XCMS program [9]. Metabolite structures were identified using precision mass-number matching (<25 ppm) and second-order map matching. After preprocessing with Pareto scaling, data were subjected to univariate statistics (Student's t test and fold change analysis) and multidimensional statistical analysis, including PCA, PLS-DA, and OPLS-DA.

## Ethics Statements

Neither human nor animal participants nor the gathering of data from social media sites were used in this study.

## CRediT authorship contribution statement

**Mai Nhu Uyen Le:** Investigation, Visualization. **Ruiqi Chen:** Validation. **Liang-e Xia:** Data curation. **Jianlin Zhou:** Supervision, Writing – original draft. **Yichong Ning:** Conceptualization, Writing – review & editing.

## Data Availability

Genome-wide identification of RUNX2 targets in osteosarcoma cells (Original data) (Gene Expression Omnibus). Effect of RUNX2 silencing on metabolomic profiles in SJSA-1 osteosarcoma cells (Original data) (China National GeneBank Database).
